# Vitamin D receptor and binding protein genes variants in patients with migraine

**DOI:** 10.1002/acn3.51872

**Published:** 2023-08-08

**Authors:** Elena García‐Martín, Santiago Navarro‐Muñoz, Pedro Ayuso, Christopher Rodríguez, Mercedes Serrador, Hortensia Alonso‐Navarro, Marisol Calleja, Silvina Espada‐Rubio, Francisco Navacerrada, Laura Turpín‐Fenoll, Marta Recio‐Bermejo, Rafael García‐Ruiz, Jorge Millán‐Pascual, José Francisco Plaza‐Nieto, Esteban García‐Albea, José A.G. Agúndez, Félix Javier Jiménez‐Jiménez

**Affiliations:** ^1^ Universidad de Extremadura, University Institute of Molecular Pathology Biomarkers Cáceres Spain; ^2^ Section of Neurology Hospital La Mancha‐Centro Alcázar de San Juan Spain; ^3^ Department of Family Medicine Hospital “Príncipe de Asturias”, Universidad de Alcalá Alcalá de Henares, Madrid Spain; ^4^ Section of Neurology Hospital Universitario del Sureste Madrid Spain; ^5^ Department of Medicine‐Neurology Universidad de Alcalá Alcalá de Henares, Madrid Spain

## Abstract

**Background/Objectives:**

Several studies have shown a relationship between vitamin D and migraine, including the association between decreased serum 25‐hydroxyvitamin D in patients with migraine and the positive effects of vitamin D supplementations in the therapy of this disease. Two single‐nucleotide variants (SNVs) *vitamin D receptor* (*VDR*) gene, *VDR* rs2228570, and *VDR* rs731236 have shown an association with migraine risk in a previous case–control association study, while an exome sequencing study identified a rare variant in *GC vitamin D binding protein* gene. This study aims to look for the association between several common variants in these two genes and the risk for migraine.

**Methods:**

We genotyped 290 patients diagnosed with migraine and 300 age‐matched controls using specific TaqMan assays for *VDR* rs2228570, *VDR* rs731236, *VDR* rs7975232, *VDR* rs739837, *VDR* rs78783628, *GC* rs7041, and *GC* rs4588 SNVs.

**Results:**

We did not find an association between these SNVs and the risk for migraine. None of these SNVs were related to the positivity of a family history of migraine or with the presence of aura. The *VDR* rs731236A allele showed a significant association with the triggering of migraine attacks by ethanol (Pc = 0.007).

**Conclusions:**

In summary, the results of the current study suggest a lack of association between common SNVs in the *VDR* and *GC* gene and the risk of developing migraine. The possible relationship between *VDR* rs731236 and the triggering of migraine episodes with ethanol deserves future studies.

## Introduction

Migraine affects 10%–18% of the population, approximately 2–3 women for each man, therefore being one of the most frequent neurological disorders. Both patients diagnosed with migraine with aura (MWA) and migraine without aura (MWoA) refer very frequently (50%–70%) positive family history of migraine and, on the other hand, individuals with first‐degree parents affected with MWA or MWoA have increased risk for both MWA and MWoA have shown an increased risk for these conditions. Despite these data suggesting an important role of genetic factors in the risk for migraine, the genetics of this disease is not completely established, except for the identification of *CACNA1A*, *ATP1A2*, and *SCN1A* genes as causative for familial hemiplegic migraine.^1^ A recent meta‐analysis of previous hypothesis‐free genome‐wide association studies (GWAS), which involved 102,084 migraine cases and 771,257 controls identified 123 susceptibility loci for migraine 86 of them previously unknown.^2^ A lot of hypothesis‐driven case–control association studies involving many candidate genes have been reported during the last 30 years, but the results of these studies regarding association with migraine have been inconsistent. It is out of the scope of the present article a detailed revision of these studies.

There are many studies describing a relationship between serum 25‐hydroxyvitamin D3 levels and migraine. Mottaghi et al.^3^ described a weak correlation of serum vitamin D with the frequency of migraine attacks but not with migraine severity. Several authors found decreased levels of serum vitamin D levels in patients with migraine compared with controls,^4^, ^5^, ^6^, ^7^ or with reference values,^8^ that was more marked^5^ or similar^6^ in patients with chronic migraine than in those with episodic migraine, and a correlation between vitamin D status and headache frequency^7^, ^8^ or severity^7^ has been described in several studies. In contrast, other authors found similar serum 25‐hydroxyvitamin D3 levels in migraine patients and controls,^9^, ^10^ and a lack of correlation of serum vitamin D status with the severity of headache.^9^ A recent meta‐analysis of previous publications confirmed decreased serum 25‐hydroxyvitamin D levels in patients with migraine compared with controls.^11^ Finally, a Mendelian randomization study involving three migraine datasets (48,975, 28,852, and 10,536 cases each) described an association between increased serum vitamin D levels and decreased risk for migraine.^12^

Buettner et al.^13^ described decreased risk for a headache of migraine type (although not with the severity of headache) in subjects with higher serum levels of vitamin D and statin use, in a series of 5938 participants. Serum vitamin D receptor levels have been found to be decreased, and serum vitamin D‐binding protein levels are similar in migraine patients compared with controls in a single study.^4^

Several randomized clinical trials have shown a beneficial effect of vitamin D supplementation in the therapy of migraine in adults^14^, ^15^, ^16^, ^17^, ^18^ and children,^19^ one of them using vitamin D in combination with simvastatin,^14^ and another showing a synergistic effect with topiramate. Two meta‐analyses of randomized clinical trials showed improvement in migraine based on a reduced number of headache days,^20^, ^21^ the frequency of headache attacks.^20^, ^21^ headache severity,^20^ and Migraine Disability Assessment Score,^20^, ^21^ but no effect in the duration of the migraine attack.^21^ In one study, patients under vitamin D therapy showed a reduction in serum levels of inducible nitric oxide synthase (iNOS) and a trend towards lower serum interleukin 6 (IL‐6) levels compared with the placebo group, while serum levels of IL‐10 and cyclooxygenase‐2 (Cox‐2) were similar in both groups.^17^ Another study showed a significant reduction in the serum calcitonin gene‐related peptide (CGRP, the most important mediator of migraine pain pathogenesis) in patients treated with vitamin D compared with those under placebo.^18^ The beneficial effect of vitamin D in migraine could be related to their effects on neuronal and immune homeostasis, and their anti‐inflammatory and antioxidant effects^22^ as inflammatory factors seem to play an important role in the pathogenesis of migraine.^23^

The nuclear hormone receptor for vitamin D3 is encoded by the *VDR* gene (link http://www.ncbi.nlm.nih.gov/gene/7421; chromosome 12q13.11; Gene ID 7421; MIM 601769), which, according to the gnomAD database, has five common single‐nucleotide variants (SNV) with frequencies higher than 0.0010 and known functional impact in Caucasians: (a) rs2228570 (*Fok1*) causes an amino acid substitution (Met 1, start loss), (b) rs731236 (*Taq1*) is a synonymous SNV not causing amino acid substitution (Ile352Ile), (c and d) rs739837, and rs78783628 are two common variants located in the 3′ untranslated area, and (e) rs7975232 (*Apa1*) is a common intronic variant.

Vitamin D binding protein, which binds to vitamin D and their plasma metabolites and acts as a transporter of vitamin D to target tissues, is encoded by the *GC vitamin D binding protein* gene (link http://www.ncbi.nlm.nih.gov/gene; chromosome 4q13.3; Gene ID 2638; MIM 139200). Two main SNVs in the *GC* gene with frequencies higher than 0.0025 and known functional impact affecting this gene have been described in Caucasians, according to the gnomAD database: (a) rs7041, which is a missense SNV causing the amino acid substitution Asp 451 Glu, and (b) and rs4588, which is another missense SNV causing the amino acid substitution Thr 455 Lys. The intronic SNV *GC* rs3755967, which has been related to vitamin D levels, is in strong linkage disequilibrium with *GC* rs4588.^24^, ^25^, ^26^

Two previous studies addressed the possible relationship between *VDR* or *GC* variants and the risk for migraine. Motaghi et al.,^27^ in a case–control association study involving 103 patients diagnosed with migraine with aura and 100 healthy controls from Iran, described an association of *VDR* rs2228570 and *VDR* rs731236 SNVs and the risk of migraine with aura. Nagata et al.^28^ identified a variant (R21L) in the exon 2 of the *GC vitamin D binding protein* gene through linkage analysis and exome sequencing in a family with four affected individuals.

The main aim of our study is to replicate the finding of Motaghi et al.^27^ In addition, we studied other common SNVs in the *VDR* and *GC* genes in Caucasian Spanish patients diagnosed with migraine and in healthy controls.

## Patients and Methods

### Patients and controls

We studied the genotype and allelic variants *VDR* rs2228570, *VDR* rs731236, *VDR* rs7975232, *VDR* rs739837, *VDR* rs78783628, *GC* rs7041, and *GC* rs4588 in 290 patients fulfilling standardized diagnostic criteria for migraine^29^ and 300 age‐ and sex‐matched controls. We recruited patients diagnosed with migraine, not suffering from other headache types or other neurological diseases, from the general neurologic clinics of several University Hospitals during 2 periods (197 between September 2006–September 2007 and 93 between June 2017 and February 2019). The healthy controls were mainly students of staff from the University of Extremadura who had neither personal nor family history and migraine and did not suffer from other types of headache), and were recruited during the same periods (215 in the first and 85 in the second ones). Most of them participated in previous case–control association genetic studies reported by our group.^23^, ^30^, ^31^, ^32^, ^33^, ^34^, ^35^, ^36^, ^37^, ^38^, ^39^, ^40^ Table 1 summarizes the demographic data of both migraine patients and control groups.

**Table 1:** Demographic and clinical data of the series studied.

Group	Migraine patients (*n* = 290)	Healthy controls (*n* = 300)
Age (years): mean (SD); range	38.8 (13.7); 13–73	38.9 (13.4); 19–77
Age at onset (years): mean (SD); range	17.8 (11.0); 2–67	NA
Age at onset < 15 years: *N* (%)	155 (53.4%)	NA
Female *N* (%)	211 (72.8)	218 (72.7)
Positive family history: *N* (%)	220 (75.9)	NA
Aura: *N* (%)	144 (49.6)	NA

NA, not available.

### Ethical aspects

The study was approved by the Ethics Committees of the University Hospital of Badajoz, University Hospital “Príncipe de Asturias” (Alcalá de Henares, Madrid, Spain), Hospital La Mancha‐Centro (Alcázar de San Juan, Ciudad Real, Spain), and the Ethics Committee of the province of Cáceres, and was performed according to the principles of the Declaration of Helsinki. After fully explaining the objectives and the procedure, participants signed informed consent to be included in the study.

### Genotyping of *VDR* rs2228570, *VDR* rs731236, *VDR* rs7975232, *VDR* rs739837, and *VDR* rs78783628, *GC* rs7041, and *GC* rs4588 variants

Genotyping of the analyzed variants was performed by using genomic DNA obtained from peripheral leukocytes of venous blood samples of both ET patients and controls, and using specific TaqMan Assays (Life Technologies, Alcobendas, Madrid, Spain) for the SNVs *VDR* rs2228570 (C__12060045_20); *VDR* rs731236 (C__2404008_10); *VDR* rs7975232 (C__28977635_10); *VDR* rs739837 (C___2404007_10); *VDR* rs78783628 (custom‐designed TaqMan probes), *GC* rs7041 (C___3133594_30); and *GC* rs4588 (C___8278879_10), as it was previously described elsewhere.^41^ The SNVs selection was done considering both their functional impact and their clinical associations, together with the allele frequencies reported in individuals of European descent according to the public database gnomAD (https://gnomad.broadinstitute.org/).

### Statistical analysis

The statistical analysis was performed by using the SPSS 27.0 version for Windows (SPSS Inc., Chicago, Illinois, USA), and the confirmation of Hardy–Weinberg equilibrium in both groups with the online program https://ihg.gsf.de/cgi‐bin/hw/hwa1.pl. The chi‐squared test, or Fisher's exact test where appropriate, were used to calculate the intergroup comparison values. We also calculated the 95% confidence intervals and the negative predictive values^42^ and used the False Discovery Rate (FDR) to perform the correction for multiple comparison adjustments.^43^

The sample size was calculated according to a genetic model that analyzed the frequency of the lower allele with an odds ratio (OR) value = 1.5 (*α* = 0.05) from the allelic frequencies found in healthy subjects. According to this sample, the statistical power (two‐tailed association) for variant alleles was, respectively, 92.83% for *VDR* rs2228570, 93.5% for *VDR* rs731236, 92.5% for *VDR* rs7975232, 92.5% for *VDR* rs739837, 93.5% for *VDR* rs78783628, 93.3% for *GC* rs7041, and 92.0% for rs4588. The Student's *t*‐test was used for the comparison of the mean ± SD age at the onset of migraine across the different genotypes of the SNVs studied.

We also analyzed the possible influence in the frequency of the genotype or allelic variants of migraine patients according to several variables which included (a)  positive or negative family history of migraine, (b) presence or absence of aura, (c) triggering or not of migraine attacks with ethanol. The chi‐squared test, Fisher's exact test, or the student *t*‐tests were used where appropriate.

The report of this study has been performed according to broad EQUATOR guidelines,^44^ specifically STROBE and STREGA checklists, which are summarized in Tables S1 and S2.

## Results

The Hardy–Weinberg equilibrium was fulfilled by the frequencies of the genotypes and allelic variants of *VDR* rs2228570, *VDR* rs731236, *VDR* rs7975232, *VDR* rs739837, *VDR* rs78783628, *GC* rs7041, and *GC* rs4588 SNVs, both in migraine patients and in controls. The frequencies of most of the genotypes and allelic variants did not differ significantly between the two groups, with the exception of a lower frequency of the *VDR* rs731236 (A/G) and *GC* rs4588 (G/T) variants, and a higher frequency of *GC* rs7041 (C/C) and *GC* rs4588 (G/G) variants in migraine patients, that disappeared after correction for multiple comparisons (Table 2). When analyzing each sex separately migraine women showed a lower frequency of the *VDR* rs731236 (A/G) genotype, which disappeared after correction for multiple comparisons as well (Table S3).

**Table 2:** *VDR* and *GC* genotypes of patients with migraine and healthy controls.

Gene	Genotype	Migraine patients (*N* = 290, 580 alleles)	Controls (*N* = 300, 600 alleles)	OR (95% CI), P; Pc; NPV (95% CI)
VDR	rs2228570 (G/G)	118 (40.7; 35.0–46.3)	122 (40.7; 35.1–46.2)	1.00 (0.72–1.39); 0.995; 0.995; 0.51 (0.47–0.54)
VDR	rs2228570 (G/A)	136 (46.9; 41.2–52.6)	137 (45.7; 40.0–51.3)	1.05 (0.76–1.45); 0.765; 0.945; 0.51 (0.48–0.55)
VDR	rs2228570 (A/A)	36 (12.4; 8.6–16.2)	41 (13.7; 9.8–17.6)	0.90 (0.55–1.45); 0.652; 0.887; 0.51 (0.49–0.52)
VDR	rs731236 (A/A)	110 (37.9; 32.3–43.5)	96 (32.0; 26.7–37.3)	1.30 (0.93–1.82); 0.131; 0.459; 0.53 (0.48–0.59)
VDR	rs731236 (A/G)	123 (42.4; 36.7–48.1)	154 (51.3; 45.7–57.0)	0.70 (0.51–0.97); **0.030**; 0.231; 0.47 (0.43–0.51)
VDR	rs731236 (G/G)	57 (19.7; 15.1–24.2)	50 (16.7; 12.4–20.9)	1.22 (0.80–1.86); 0.347; 0.685; 0.52 (0.50–0.54)
VDR	rs7975232 (C/C)	66 (22.8; 17.9–27.6)	59 (19.7; 15.2–24.2)	1.20 (0.81–1.79); 0.359; 0.685; 0.52 (0.50–0.54)
VDR	rs7975232 (C/A)	132 (45.5; 39.8–51.2)	147 (49.0; 43.3–54.7)	0.87 (0.53–1.20); 0.397; 0.695; 0.49 (0.45–0.53)
VDR	rs7975232 (A/A)	92 (31.7; 26.4–37.1)	94 (31.3; 26.1–36.6)	1.02 (0.72–1.44); 0.919; 0.995; 0.51 (0.48–0.54)
VDR	rs739837 (G/G)	67 (23.1; 18.3–28.0)	57 (19.0; 14.6–23.4)	1.28 (0.86–1.91); 0.222; 0.666; 0.52 (0.46–0.62)
VDR	rs739837 (G/T)	134 (46.2; 40.5–51.9)	151 (50.3; 44.7–56.0)	0.85 (0.61–1.17); 0.316; 0.685; 0.49 (0.45–0.53)
VDR	rs739837 (T/T)	89 (30.7; 25.4–36.0)	92 (30.7; 25.4–35.9)	1.00 (0.71–1.42); 0.995; 0.995; 0.51 (0.48–0.54)
VDR	rs78783628 (A/A)	91 (31.4; 26.0–36.7)	87 (29.0; 23.9–34.1)	1.12 (0.79–1.59); 0.529; 0.854; 0.52 (0.49–0.54)
VDR	rs78783628 (A/−)	132 (45.5; 39.8–51.2)	148 (49.3; 43.7–55.0)	0.86 (0.62–1.19); 0.354; 0.685; 0.49 (0.45–0.53)
VDR	rs78783628 (−/−)	67 (23.1; 18.3–28.0)	65 (21.7; 17.0–26.3)	1.09 (0.74–1.60); 0.676; 0.887; 0.51 (0.49–0.54)
GC	rs7041 (A/A)	64 (22.1; 17.3–26.8)	67 (22.3; 17.6–27.0)	0.99 (0.67–1.45); 0.938; 0.995; 0.51 (0.49–0.53)
GC	rs7041 (A/C)	137 (47.2; 41.5–53.0)	163 (54.3; 48.7–60.0)	0.75 (0.55–1.04); 0.085; 0.357; 0.48 (0.43–0.52)
GC	rs7041 (C/C)	89 (30.7; 25.4–36.0)	70 (23.3; 18.5–28.1)	1.46 (1.01–2.10); **0.044**; 0.231; 0.53 (0.51–0.56)
GC	rs4588 (G/G)	149 (51.4; 45.6–57.1)	129 (43.0; 37.4–48.6)	1.40 (1.01–1.94); **0.042**; 0.231; 0.55 (0.51–0.59)
GC	rs4588 (G/T)	112 (38.6; 33.0–44.2)	145 (48.3; 42.7–54.0)	0.67 (0.49–0.93); **0.017**; 0.231; 0.47 (0.43–0.50)
GC	rs4588 (T/T)	29 (10.0; 6.5–13.5)	26 (8.7; 5.5–11.9)	1.17 (0.67–2.04); 0.578; 0.867; 0.51 (0.50–0.53)

	Alleles	Migraine patients (*N* = 290, 580 alleles)	Controls (*N* = 300, 600 alleles)	OR (95% CI), P; Pc; NPV (95% CI)
VDR	rs2228570 (G)	372 (64.1; 60.2–68.0)	381 (63.5; 59.6–67.4)	1.03 (0.81–1.30); 0.820; 0.871; 0.51 (0.47–0.55)
VDR	rs2228570 (A)	208 (35.9; 32.0–39.8)	219 (36.5; 32.6–40.4)	0.97 (0.77–1.23); 0.820; 0.871; 0.51 (0.48–0.53)
VDR	rs731236 (A)	343 (59.1; 55.1–63.1)	346 (57.7; 53.7–61.6)	1.06 (0.84–1.34); 0.608; 0.871; 0.52 (0.48–0.55)
VDR	rs731236 (G)	237 (40.9; 36.9–44.9)	254 (42.3; 38.4–46.3)	0.94 (0.75–1.19); 0.608; 0.871; 0.50 (0.48–0.53)
VDR	rs7975232 (C)	264 (45.5; 41.5–49.6)	265 (44.2; 40.2–48.1)	1.06 (0.84–1.33); 0.641; 0.871;0.52 (0.49–0.54)
VDR	rs7975232 (A)	316 (54.5; 50.4–58.5)	335 (55.8; 51.9–59.8)	0.95 (0.75–1.19); 0.641; 0.871; 0.50 (0.47–0.53)
VDR	rs739837 (G)	268 (46.2; 42.1–50.3)	265 (44.2; 40.2–48.1)	1.09 (0.86–1.37); 0.482; 0.871;0.52 (0.49–0.54)
VDR	rs739837 (T)	312 (53.8; 49.7–57.9)	335 (55.8; 51.9–59.8)	0.92 (0.73–1.16); 0.482; 0.871;0.50 (0.47–0.53)
VDR	rs78783628 (A)	314 (54.1; 50.1–58.2)	322 (53.7; 49.7–57.7)	1.02 (0.81–1.28); 0.871; 0.871;0.51 (0.48–0.54)
VDR	rs78783628 (−)	266 (45.9; 41.8–49.9)	278 (46.3; 42.3–50.3)	0.98 (0.78–1.23); 0.871; 0.871;0.51 (0.48–0.53)
GC	rs7041 (A)	265 (45.7; 41.6–49.7)	297 (49.5; 45.5–53.5)	0.86 (0.68–1.08); 0.190; 0.669; 0.49 (0.46–0.52)
GC	rs7041 (C)	315 (54.3; 50.3–58.4)	303 (50.5; 46.5–54.5)	1.17 (0.93–1.47); 0.190; 0.669; 0.53 (0.50–0.56)
GC	rs4588 (G)	410 (70.7; 67.0–74.4)	403 (67.2; 63.4–70.9)	1.18 (0.92–1.51); 0.191; 0.669; 0.54 (0.49–0.58)
GC	rs4588 (T)	170 (29.3; 25.6–33.0)	197 (32.8; 29.1–36.6)	0.85 (0.66–1.09); 0.191; 0.669; 0.50 (0.48–0.52)

Test for trend for variant alleles that presented statistically significant crude *p* values: rs731236: OR 0.97; chi‐squared 0.25; *p* = 0.614. rs7041: OR 1.16; chi‐squared 1.75; *p* = 0.186. rs4588: OR 0.90; chi‐squared 1.74; *p* = 0.188. Bold values indicate statistically significant of *p*‐values.

Mean ± SD age at onset of migraine attacks was significantly lower in patients carrying the *VDR* rs731236 (G/G) genotype compared with those carrying the *VDR* rs731236 (A/G) genotype and in those carrying the *GC* rs7041 (A/C) compared with *GC* rs7041 (A/A) genotype (Table 3).

**Table 3:** Age at onset of migraine according to the *VDR* and *GC* genotypes.

Gene	Genotype	Age at onset (mean ± SD)	*t*‐test compared to the non‐mutated genotype (*p*‐value)	*t*‐test compared to the heterozygous genotype (*p*‐value)
VDR	rs2228570 (G/G)	17.89 ± 10.43		
VDR	rs2228570 (G/A)	17.68 ± 10.88	0.878	
VDR	rs2228570 (A/A)	18.14 ± 11.88	0.904	0.827
VDR	rs731236 (A/A)	17.75 ± 10.36		
VDR	rs731236 (A/G)	19.07 ± 11.44	0.363	
VDR	rs731236 (G/G)	15.28 ± 9.84	0.139	**0.033**
VDR	rs7975232 (C/C)	17.30 ± 10.06		
VDR	rs7975232 (C/A)	18.51 ± 10.63	0.445	
VDR	rs7975232 (A/A)	17.22 ± 11.56	0.961	0.390
VDR	rs739837 (G/G)	16.94 ± 10.20		
VDR	rs739837 (G/T)	18.41 ± 10.60	0.349	
VDR	rs739837 (T/T)	17.61 ± 11.55	0.708	0.593
VDR	rs78783628 (A/A)	17.47 ± 11.47		
VDR	rs78783628 (A/−)	18.52 ± 10.64	0.487	
VDR	rs78783628 (−/−)	16.94 ± 10.20	0.763	0.318
GC	rs7041 (A/A)	19.84 ± 11.74		
GC	rs7041 (A/C)	16.74 ± 9.23	**0.043**	
GC	rs7041 (C/C)	18.04 ± 12.14	0.361	0.360
GC	rs4588 (G/G)	17.96 ± 11.37		
GC	rs4588 (G/T)	17.21 ± 9.26	0.571	
GC	rs4588 (T/T)	19.48 ± 13.20	0.521	0.287

Bold values indicate statistically significant of *p*‐values.

The genotype and allele frequencies in patients with migraine were similar in patients with versus without positive family history of migraine (Table S4), and in patients with migraine with aura compared with those with migraine without aura (Table S4). The frequencies of *VDR* rs731236 (A/A) and *VDR* rs731236 (G/G) genotypes were, respectively, significantly higher and significantly lower in patients in which alcohol was a triggering factor for migraine attacks (Table S4). Despite these genotype differences disappearing after correction for multiple comparisons, the allele frequencies were different even after correction for multiple comparisons (Pc = 0.007; Table S4), and the test for trend for variant alleles showed a significant association of the *VDR* rs731236 SNV with the ethanol effect, with an OR = 0.58 (*p* = 0.0016).

## Discussion

The decreased serum 25‐hydroxyvitamin levels found in patients with migraine, together with the described beneficial effects of vitamin D in this disease make it reasonable to investigate the possible association between SNVs in vitamin D‐related genes and the risk for migraine. While Motaghi et al.^27^ described the association of *VDR* rs2228570 and *VDR* rs731236 SNVs with the risk of migraine with aura, data from the current study, did not confirm an association of the five analyzed SNVs in the *VDR* gene with the risk for migraine, with or without aura, and we did not find association either in patients with or those without a family history of migraine. In addition, we did not find an association between the two most common SNVs in the *GC vitamin D binding protein* gene and migraine risk. On the other hand, subjects carrying the minor allele of *VDR* rs731236 SNV showed a lower frequency of induction of migraine attacks by ethanol, being this finding difficult to explain.

Association between SNVs in the *VDR* and *GC vitamin D‐binding protein* genes and the risk for other neurological diseases has been a matter of several studies. *VDR* rs2228570^45^, ^46^ and *VDR* rs7975232^45^ have been associated with the risk for Parkinson's disease, at least in the Asian population,^46^ *VDR* rs731236 with the risk for Alzheimer's disease,^45^, ^46^ at least in Caucasians,^47^ and *VDR* rs1544410 and *VDR* rs7975232, respectively, with increased and decreased risk for mild cognitive impairment.^47^ Pooled data of two studies^48^, ^49^ showed an association between *VDR* rs2228570 and essential tremor, while *VDR* rs731236,^49^, ^50^ *VDR* rs7975232,^49^ *VDR* rs739837,^49^ and *VDR* rs78783628^49^ showed lack of association with this disease. Other described associations of SNVs in the *VDR* gene include an association of *VDR* rs7975232 and the risk for multiple sclerosis^51^ and amyotrophic lateral sclerosis,^52^ *VDR* rs2228570 with ischemic stroke,^53^ *VDR* rs2228570 and *VDR* rs7975232 with childhood temporal lobe epilepsy,^54^ and *VDR* rs731236 with adult non‐thymoma myasthenia gravis with the negativity of acetylcholine receptors antibodies.^55^ An association of *VDR* rs2228570 and *VDR* rs731236 with the risk for restless legs syndrome reported in one study^41^ was not confirmed in another.^56^

*GC* rs7041 SNV has been associated with the risk for Parkinson's disease,^57^ while *GC* rs7041, *GC* rs4588, and *GC* rs2282679 were not associated with the risk for multiple sclerosis,^58^ and *GC* rs7041and *GC* rs4588 were not associated to restless legs syndrome.^56^

Taking into account the relatively low sample size as the main limitation of the current study, our results suggest the lack of association of the most common SNVs in the two genes analyzed with the risk for migraine in the Caucasian Spanish population. The finding on the possible influence of the *VDR* rs731236 SNV in triggering migraine attacks by ethanol deserves further studies.

## Author Contributions

All authors fulfill the criteria of authorship and no one else who fulfills the criteria has been excluded. All of them have approved the final submitted version. EGM: Drafting/revising the manuscript for content, including medical writing for content; study concept or design; acquisition of data; interpretation of data; study supervision and coordination, obtaining funding. SNM: Drafting/revising the manuscript for content, including medical writing for content; acquisition of data. PA: Drafting/revising the manuscript for content, including medical writing for content; acquisition of data. CR: Drafting/revising the manuscript for content, including medical writing for content; acquisition of data. MS: Drafting/revising the manuscript for content, including medical writing for content; acquisition of data. HAN: Drafting/revising the manuscript for content, including medical writing for content; study concept or design; acquisition of data; interpretation of data; study supervision and coordination. MC: Drafting/revising the manuscript for content, including medical writing for content; acquisition of data. SER: Drafting/revising the manuscript for content, including medical writing for content; acquisition of data. FN: Drafting/revising the manuscript for content, including medical writing for content; acquisition of data. LTF: Drafting/revising the manuscript for content, including medical writing for content; acquisition of data. MRB: Drafting/revising the manuscript for content, including medical writing for content; acquisition of data. RGR: Drafting/revising the manuscript for content, including medical writing for content; acquisition of data. JMP: Drafting/revising the manuscript for content, including medical writing for content; acquisition of data. JFPN: Drafting/revising the manuscript for content, including medical writing for content; acquisition of data. EGA: Drafting/revising the manuscript for content, including medical writing for content; acquisition of data. JAGA: Drafting/revising the manuscript for content, including medical writing for content; study concept or design; acquisition of data; statistical analysis and interpretation of data; study supervision and coordination, obtaining funding. FJJJ: Drafting/revising the manuscript for content, including medical writing for content; study concept or design; acquisition of data; analysis or interpretation of data; study supervision and coordination.

## Funding Information

This work was supported in part by, PI18/00540 and PI21/01683 from Fondo de Investigación Sanitaria, Instituto de Salud Carlos III, Madrid, Spain, and IB20134 and GR21073 from Junta de Extremadura, Mérida, Spain. Partially funded with FEDER funds.

## Conflict of Interest Statement

All authors declare that there is no financial or nonfinancial conflict of interest.

## Supporting information


Table S1.
Table S2.
Table S3.
Table S4.

## Data Availability

All data related to the current study, intended for reasonable use, is available from J.A.G. Agúndez (University Institute of Molecular Pathology Biomarkers, University of Extremadura ‐UNEx ARADyAL Instituto de Salud Carlos III, Av/ de la Universidad S/N, E10071 Cáceres. Spain) and F.J. Jiménez‐Jiménez (Section of Neurology, Hospital del Sureste, Arganda del Rey, Madrid, Spain).

## References

[1] Mateos V , Pareja JA , Pascual J . Tratado de cefaleas. Luzán 5 S.A. Ediciones; 2009.

[2] Hautakangas H , Winsvold BS , Ruotsalainen SE , et al. Genome‐wide analysis of 102,084 migraine cases identifies 123 risk loci and subtype‐specific risk alleles. Nat Genet. 2022;54:152‐160. doi:10.1038/s41588-021-00990-0 35115687 PMC8837554

[3] Mottaghi T , Khorvash F , Askari G , et al. The relationship between serum levels of vitamin D and migraine. J Res Med Sci. 2013;18(Suppl 1):S66‐S70. 23961291 PMC3743325

[4] Celikbilek A , Gocmen AY , Zararsiz G , et al. Serum levels of vitamin D, vitamin D‐binding protein and vitamin D receptor in migraine patients from Central Anatolia region. Int J Clin Pract. 2014;68(10):1272‐1277. doi:10.1111/ijcp.12456 24837712

[5] Rapisarda L , Mazza MR , Tosto F , Gambardella A , Bono F , Sarica A . Relationship between severity of migraine and vitamin D deficiency: a case‐control study. Neurol Sci. 2018;39(Suppl 1):167‐168. doi:10.1007/s10072-018-3384-4 29904877

[6] Togha M , Razeghi Jahromi S , Ghorbani Z , Martami F , Seifishahpar M . Serum vitamin D status in a group of migraine patients compared with healthy controls: a case‐control study. Headache. 2018;58(10):1530‐1540. doi:10.1111/head.13423 30341768

[7] Hussein M , Fathy W , Abd Elkareem RM . The potential role of serum vitamin D level in migraine headache: a case‐control study. J Pain Res. 2019;12:2529‐2536. doi:10.2147/JPR.S216314 31686895 PMC6709376

[8] Song TJ , Chu MK , Sohn JH , Ahn HY , Lee SH , Cho SJ . Effect of vitamin D deficiency on the frequency of headaches in migraine. J Clin Neurol. 2018;14(3):366‐373. doi:10.3988/jcn.2018.14.3.366 29971976 PMC6031995

[9] Zandifar A , Masjedi SS , Banihashemi M , et al. Vitamin D status in migraine patients: a case‐control study. Biomed Res Int. 2014;2014:514782. doi:10.1155/2014/514782 24524078 PMC3910019

[10] Kjaergaard M , Eggen AE , Mathiesen EB , Jorde R . Association between headache and serum 25‐hydroxyvitamin D: the Tromsø study: Tromsø 6. Headache. 2012;52(10):1499‐1505. doi:10.1111/j.1526-4610.2012.02250.x 22973803

[11] Liampas I , Siokas V , Brotis A , Dardiotis E . Vitamin D serum levels in patients with migraine: a meta‐analysis. Rev Neurol (Paris). 2020;176(7–8):560‐570. doi:10.1016/j.neurol.2019.12.008 32241571

[12] Niu PP , Wang X , Xu YM . Higher circulating vitamin D levels are associated with decreased migraine risk: a Mendelian randomization study. Front Nutr. 2022;9:907789. doi:10.3389/fnut.2022.907789 36159496 PMC9505695

[13] Buettner C , Burstein R . Association of statin use and risk for severe headache or migraine by serum vitamin D status: a cross‐sectional population‐based study. Cephalalgia. 2015;35(9):757‐766. doi:10.1177/0333102414559733 25424706

[14] Buettner C , Nir RR , Bertisch SM , et al. Simvastatin and vitamin D for migraine prevention: a randomized, controlled trial. Ann Neurol. 2015;78(6):970‐981. doi:10.1002/ana.24534 26418341 PMC4715556

[15] Mottaghi T , Askari G , Khorvash F , Maracy MR . Effect of vitamin D supplementation on symptoms and C‐reactive protein in migraine patients. J Res Med Sci. 2015;20(5):477‐482. doi:10.4103/1735-1995.163971 26487877 PMC4590203

[16] Gazerani P , Fuglsang R , Pedersen JG , et al. A randomized, double‐blinded, placebo‐controlled, parallel trial of vitamin D_3_ supplementation in adult patients with migraine. Curr Med Res Opin. 2019;35(4):715‐723. doi:10.1080/03007995.2018.1519503 30182753

[17] Ghorbani Z , Togha M , Rafiee P , et al. Vitamin D3 might improve headache characteristics and protect against inflammation in migraine: a randomized clinical trial. Neurol Sci. 2020;41(5):1183‐1192. doi:10.1007/s10072-019-04220-8 31897949

[18] Ghorbani Z , Rafiee P , Fotouhi A , et al. The effects of vitamin D supplementation on interictal serum levels of calcitonin gene‐related peptide (CGRP) in episodic migraine patients: post hoc analysis of a randomized double‐blind placebo‐controlled trial. J Headache Pain. 2020;21(1):22. doi:10.1186/s10194-020-01090-w 32093657 PMC7041277

[19] Fallah R , Sarraf Yazd S , Sohrevardi SM . Efficacy of Topiramate alone and Topiramate plus vitamin D3 in the prophylaxis of pediatric migraine: a randomized clinical trial. Iran J Child Neurol. 2020;14(4):77‐86. PMC7660031 33193786

[20] Zhang YF , Xu ZQ , Zhou HJ , Liu YZ , Jiang XJ . The efficacy of vitamin D supplementation for migraine: a meta‐analysis of randomized controlled studies. Clin Neuropharmacol. 2021;44(1):5‐8. doi:10.1097/WNF.0000000000000419 33449474

[21] Hu C , Fan Y , Wu S , Zou Y , Qu X . Vitamin D supplementation for the treatment of migraine: a meta‐analysis of randomized controlled studies. Am J Emerg Med. 2021;50:784‐788. doi:10.1016/j.ajem.2021.07.062 34879503

[22] Berretta M , Quagliariello V , Bignucolo A , et al. The multiple effects of vitamin D against chronic diseases: from reduction of lipid peroxidation to updated evidence from clinical studies. Antioxidants (Basel). 2022;11(6):1090. doi:10.3390/antiox11061090 35739987 PMC9220017

[23] García‐Martín E , Navarro‐Muñoz S , Ayuso P , et al. Lack of association between common *LAG3/CD4* variants and risk of migraine. Int J Mol Sci. 2023;24(2):1292. doi:10.3390/ijms24021292 36674807 PMC9866744

[24] Jiang X , O'Reilly PF , Aschard H , et al. Genome‐wide association study in 79,366 European‐ancestry individuals informs the genetic architecture of 25‐hydroxyvitamin D levels. Nat Commun. 2018;9:260. doi:10.1038/s41467-017-02662-2 29343764 PMC5772647

[25] Revez JA , Lin T , Qiao Z , et al. Genome‐wide association study identifies 143 loci associated with 25 hydroxyvitamin D concentration. Nat Commun. 2020;11:1647. doi:10.1038/s41467-020-15421-7 32242144 PMC7118120

[26] Manousaki D , Mitchell R , Dudding T , et al. Genome‐wide association study for vitamin D levels reveals 69 independent loci. Am J Hum Genet. 2020;106:327‐337. doi:10.1016/j.ajhg.2020.01.017 32059762 PMC7058824

[27] Motaghi M , Haghjooy Javanmard S , Haghdoost F , et al. Relationship between vitamin D receptor gene polymorphisms and migraine without aura in an Iranian population. Biomed Res Int. 2013;2013:351942. 23984350 10.1155/2013/351942 PMC3741896

[28] Nagata E , Fujii N , Hosomichi K , et al. Possible association between dysfunction of vitamin D binding protein (GC globulin) and migraine attacks. PloS One. 2014;9(8):e105319. doi:10.1371/journal.pone.0105319 25147936 PMC4141767

[29] Headache Classification Committee of the International Headache Society . The international classification of headache disorders, 2nd ed. Cephalalgia. 2004;24(Suppl. 1):1‐160. 10.1111/j.1468-2982.2003.00824.x 14979299

[30] García‐Martín E , Martínez C , Serrador M , et al. Histamine‐N‐methyl transferase polymorphism and risk for migraine. Headache. 2008;48(9):1343‐1348. doi:10.1111/j.1526-4610.2007.01056.x 18266724

[31] García‐Martín E , Martínez C , Serrador M , et al. Alcohol dehydrogenase 2 genotype and risk for migraine. Headache. 2010;50(1):85‐91. doi:10.1111/j.1526-4610.2009.01396.x 19486361

[32] García‐Martín E , Martínez C , Serrador M , et al. Dopamine receptor 3 (DRD3) polymorphism and risk for migraine. Eur J Neurol. 2010;17(9):1220‐1223. doi:10.1111/j.1468-1331.2010.02988.x 20236178

[33] García‐Martín E , Martínez C , Serrador M , et al. Paraoxonase 1 (PON1) polymorphisms and risk for migraine. J Neurol. 2010;257(9):1482‐1485. doi:10.1007/s00415-010-5551-2 20407783

[34] García‐Martín E , Martínez C , Serrador M , et al. SLC1A2 rs3794087 variant and risk for migraine. J Neurol Sci. 2014;338(1–2):92‐95. doi:10.1016/j.jns.2013.12.022 24412224

[35] García‐Martín E , Martínez C , Serrador M , et al. Diamine oxidase rs10156191 and rs2052129 variants are associated with the risk for migraine. Headache. 2015;55(2):276‐286. doi:10.1111/head.12493 25612138

[36] García‐Martín E , Martínez C , Serrador M , et al. Neuronal nitric oxide synthase (nNOS, NOS1) rs693534 and rs7977109 variants and risk for migraine. Headache. 2015;55(9):1209‐1217. doi:10.1111/head.12617 26283425

[37] García‐Martín E , Martínez C , Serrador M , et al. Gamma‐aminobutyric acid (Gaba) receptors rho (Gabrr) gene polymorphisms and risk for migraine. Headache. 2017;57(7):1118‐1135. doi:10.1111/head.13122 28699326

[38] García‐Martín E , Esguevillas G , Serrador M , et al. Gamma‐aminobutyric acid (GABA) receptors GABRA4, GABRE, and GABRQ gene polymorphisms and risk for migraine. J Neural Transm (Vienna). 2018;125(4):689‐698. doi:10.1007/s00702-017-1834-4 29299688

[39] García‐Martín E , Navarro‐Muñoz S , Rodriguez C , et al. Association between endothelial nitric oxide synthase (NOS3) rs2070744 and the risk for migraine. Pharmacogenomics J. 2020;20(3):426‐432. doi:10.1038/s41397-019-0133-x 31792366

[40] García‐Martín E , Navarro‐Muñoz S , Amo G , et al. Increased serum diamine oxidase activity in nonallergic patients with migraine. Eur J Clin Invest. 2022;52(6):e13757. doi:10.1111/eci.13757 35113457

[41] Jiménez‐Jiménez FJ , García‐Martín E , Alonso‐Navarro H , et al. Association between vitamin D receptor rs731236 (Taq1) polymorphism and risk for restless legs syndrome in the Spanish Caucasian population. Medicine (Baltimore). 2015;94(47):e2125. doi:10.1097/MD.0000000000002125 26632733 PMC5059002

[42] Altman DG , Bland JM . Diagnostic tests 2: predictive values. BMJ. 1994;309(6947):102. doi:10.1136/bmj.309.6947.102 8038641 PMC2540558

[43] Benjamini Y , Hochberg Y . Controlling the false discovery rate: a practical and powerful approach to multiple testing. J Roy Statis Soc Ser B. 1995;57(1):289‐300.

[44] Simera I , Moher D , Hoey J , Schulz KF , Altman DG . A catalogue of reporting guidelines for health research. Eur J Clin Invest. 2010;40(1):35‐53. 20055895 10.1111/j.1365-2362.2009.02234.x

[45] Geng J , Zhang J , Yao F , Liu X , Liu J , Huang Y . A systematic review and meta‐analysis of the associations of vitamin D receptor genetic variants with two types of most common neurodegenerative disorders. Aging Clin Exp Res. 2020;32(1):21‐27. doi:10.1007/s40520-019-01135-4 30863943

[46] Gao J , Teng J , Liu Z , Cai M , Xie A . Association between vitamin D receptor polymorphisms and susceptibility to Parkinson's disease: an updated meta‐analysis. Neurosci Lett. 2020;720:134778. doi:10.1016/j.neulet.2020.134778 31978499

[47] Liu N , Zhang T , Ma L , et al. Vitamin D receptor gene polymorphisms and risk of Alzheimer disease and mild cognitive impairment: a systematic review and meta‐analysis. Adv Nutr. 2021;24:nmab074. doi:10.1093/advances/nmab074 PMC8634528 34167149

[48] Sazci A , Uren N , Idrisoglu HA , Ergul E . The rs2228570 variant of the vitamin D receptor gene is associated with essential tremor. Neurosci Bull. 2019;35(2):362‐364. doi:10.1007/s12264-018-0287-6 30225763 PMC6426913

[49] Agúndez JAG , García‐Martín E , Alonso‐Navarro H , et al. Vitamin D receptor and binding protein gene variants in patients with essential tremor. Mol Neurobiol. 2022;59(6):3458‐3466. doi:10.1007/s12035-022-02804-8 35322382

[50] Chen J , Huang P , He Y , et al. IL1B polymorphism is associated with essential tremor in Chinese population. BMC Neurol. 2019;19(1):99. doi:10.1186/s12883-019-1331-5 31092216 PMC6518722

[51] Mohammadi A , Azarnezhad A , Khanbabaei H , et al. Vitamin D receptor genetic polymorphisms and the risk of multiple sclerosis: a systematic review and meta‐analysis. Steroids. 2020;158:108615. doi:10.1016/j.steroids.2020.108615 32097613

[52] Török N , Török R , Klivényi P , Engelhardt J , Vécsei L . Investigation of vitamin D receptor polymorphisms in amyotrophic lateral sclerosis. Acta Neurol Scand. 2016;133(4):302‐308. doi:10.1111/ane.12463 26190642

[53] Prabhakar P , Majumdar V , Kulkarni GB , Christopher R . Genetic variants of vitamin D receptor and susceptibility to ischemic stroke. Biochem Biophys Res Commun. 2015;456(2):631‐636. doi:10.1155/2013/351942 25498546

[54] Jiang P , Zhu WY , He X , et al. Association between vitamin D receptor gene polymorphisms with childhood temporal lobe epilepsy. Int J Environ Res Public Health. 2015;12(11):13913‐13922. doi:10.3390/ijerph121113913 26528998 PMC4661623

[55] Han JL , Yue YX , Gao X , et al. Vitamin D receptor polymorphism and myasthenia gravis in Chinese Han population. Front Neurol. 2021;12:604052. doi:10.3389/fneur.2021.604052 33633666 PMC7900549

[56] Jiménez‐Jiménez FJ , Amo G , Alonso‐Navarro H , et al. Serum vitamin D, vitamin D receptor and binding protein genes polymorphisms in restless legs syndrome. J Neurol. 2021;268(4):1461‐1472. doi:10.1007/s00415-020-10312-9 33219423

[57] Gezen‐Ak D , Alaylıoğlu M , Genç G , et al. GC and VDR SNPs and vitamin D levels in Parkinson's disease: the relevance to clinical features. Neuromolecular Med. 2017;19(1):24‐40. doi:10.1007/s12017-016-8415-9 27282160

[58] Agliardi C , Guerini FR , Zanzottera M , Bolognesi E , Costa AS , Clerici M . Vitamin D‐binding protein gene polymorphisms are not associated with MS risk in an Italian cohort. J Neuroimmunol. 2017;305:92‐95. doi:10.1016/j.jneuroim.2017.02.009 28284354

